# Burden of health‐care costs for patients with heparin replacement for colorectal EMR in Japan

**DOI:** 10.1002/prp2.366

**Published:** 2017-11-06

**Authors:** Shiro Hayashi, Dai Nakamatsu, Tokuhiro Matsubara, Masashi Yamamoto, Tsutomu Nishida

**Affiliations:** ^1^ Department of Gastroenterology and Hepatology Toyonaka municipal hospital Osaka Japan

Endoscopic mucosal resection (EMR) for colorectal polyps is a safe and well‐known procedure to prevent developing colorectal cancer. There is, however, still higher risk of post‐procedural bleeding (PPB) when patients take antithrombotic agents, especially taking dual antiplatelet therapy (DAPT) or anticoagulant. The Japanese guideline in 2012 recommends that warfarin or dabigatran should be replaced with unfractionated heparin for high‐bleeding‐risk endoscopic procedures in patients at high thrombotic risk.^1^ Recently, there are some reports about adverse effects of heparin replacement therapy (HR), which increases the risk of PPB with a pooled odds ratio of 8 up to 17.^2^, ^3^, ^4^ And furthermore, little has been reported yet about other disadvantages of HR such as longer hospital stays or costs of hospitalization and high incidence of PPB.

We therefore evaluated the clinical impacts of cost‐balance of colorectal EMR between HR group and non‐HR group in Japan.

We enrolled 59 consecutive patients who underwent colorectal EMR with HR at our institution between March 2015 and April 2016. We evaluated the incidence of PPB, taking anticoagulants or antiplatelets, duration of hospital stay, and costs of hospitalization based on the database of the diagnosis procedure combination (DPC)‐based payment system in Japan. A total of 59 patients [64% men; mean age, 71 ± 11 years] were evaluated. With regard to anticoagulant agents, 30 patients (51%) were taking warfarin and 29 patients (49%) were taking direct oral anticoagulant (DOAC). The types of DOACs were dabigatran (7, 24%), rivaroxaban (16, 55%), apixaban (5, 17%), and edoxaban (1, 3.4%). The causes of taking anticoagulants were atrial fibrillation (48, 81%), heart valve replacement (3, 5.0%), arteriosclerosis obliterans (3, 5.0%), deep venous thrombosis (2, 3.4%), and the others (3, 5.0%). With regard to antiplatelet agents, 15 patients (25%) were taking single antiplatelet agent (APA), two patients (3.4%) were taking DAPT, and one patient (1.7%) was taking triplet agents. EMR was performed on 12 patients (20%) under HR with continuing low‐dose aspirin. The overall mean hospital days were 9.5 (3–34) days. PPB occurred in six patients (10%) who required endoscopic hemostasis. No thromboembolic event was observed. The overall mean cost was 324,343 ± 176,210 yen (about 2,975 ± 1,616 dollars). On the other hand, the cost without HR for a 3‐day hospital stay calculated from DPC payment system was 148,746 yen (about 1,365 dollar). The breakdown of anticoagulants showed that PPB was 6.7% (2/30) in warfarin and 13.8% (4/29) in DOACs (*P *=* *0.42), the mean hospital days was 13.4 vs 5.3 (*P *<* *0.0001) and mean cost was 445,910 vs 198,301 yen (4,091 vs 1,819 dollar, *P *<* *0.0001), respectively. With regard to APA, the incidence of PPB were 33% (4/12) in continuing group and 4.3% (2/47) in discontinuing group (*P *=* *0.0127). We found that colorectal EMR under HR doubled the cost and more than tripled the hospital stay than standard treatment. Moreover, continuing APA under HR caused higher PPB risk. Warfarin tended to lower PPB than DOACs but higher cost due to longer hospital stay. Because of both the reasons of high bleeding risk and high cost, heparin replacement therapy may be replaced by other strategy.
